# Chronic unilateral tearing as a sign of lacrimal sac squamous cell carcinoma

**DOI:** 10.5935/0004-2749.20210099

**Published:** 2025-08-21

**Authors:** Aline Mota Freitas Matos, Danielle Ribeiro Brega, Leonardo Provetti Cunha, Angela Maria Gollner, Allan Christian Pieroni Gonçalves

**Affiliations:** 1 Department of Ophthalmology, Hospital Universitário, Universidade Federal de Juiz de Fora, Juiz de Fora, MG, Brazil; 2 Division of Ophthalmology, Faculdade de Medicina, Universidade de São Paulo, São Paulo SP, Brazil; 3 Department of Pathology, Hospital Universitário, Universidade Federal de Juiz de Fora, Juiz de Fora, MG, Brazil; 4 Division of Ophthalmology, Faculdade de Medicina do ABC, São Paulo SP, Brazil

**Keywords:** Lacrimal duct obstruction, Lacrimal apparatus diseases, Eye neoplasms, Carcinoma, squamous cell, Magnetic resonance imaging, Tomography, x-ray computed, Human, Case reports, Obstrução dos ductos lacrimais, Doenças do aparelho lacrimal, Neoplasias oculares, Carcinoma de células escamosas, Imagem por ressonância magnética, Tomografia computadorizada por raios X, Humano, Relato de casos

## Abstract

Tearing is a very common symptom in ophthalmic practice; however, this issue is commonly overlooked. The authors describe the case of a patient with chronic unilateral epiphora that had been neglected for 8 y; lacrimal system workup was performed for this patient only because of dacryocystitis evolvement. Following the diagnosis of nasolacrimal duct blockage, dacryocystorhinostomy was indicated. Intraoperative abnormal mucosa was subjected to biopsy, and a diagnosis of squamous cell carcinoma in the lacrimal sac was established. Extended tumor excision was performed for the patient and adjuvant radiotherapy was administered, without any recurrence till the 2-year follow-up. The present report highlights the relevance of a detailed evaluation for determining the underlying causes of tearing, especially in chronic unilateral presentation. The consideration of potential malignancy in such cases could prevent delayed diagnosis of uncommon but potentially life-threatening malignancies of the lacrimal drainage system.

## Abstract (pt)

O lacrimejamento é um sinal comum na prática oftalmológica, porém frequentemente negligenciado. Os autores descrevem um caso de um paciente com epífora unilateral direita com 8 anos de história que só após evoluir com dacriocistite foi submetido a semiologia de vias lacrimais. Após o diagnóstico de obstrução baixa da via lacrimal foi submetido a cirurgia de dacriocistorrinostomia, onde o saco lacrimal de aparência atípica foi biopsiado e identificado como carcinoma espinocelular. O paciente foi submetido a ressecção oncológica do tumor seguido de radioterapia adjuvante, permanecendo livre de recidiva por 2 anos. Este caso ressalta a importância de investigar causas da epífora, especialmente quando unilateral, dada sua maior associação com a obstrução de via lacrimal. Uma semiologia oportuna das vias lacrimais podem evitar atrasos no diagnóstico de obstrução secundárias a neoplasia, que apesar de incomuns, são potencialmente fatais.

## INTRODUCTION

Excessive tearing or epiphora in adults is a common complaint by most patients who visit an ophthalmologist; however, this complaint is commonly ignored. This condition has several etiologies, and there is no consensus on the optimal method of evaluation. In the absence of dry eye symptoms, inflammation, or conjunctival hyperemia, a tear outflow abnormality should be considered. Reduced tear drainage may occur because of eyelid malposition, tear pump dysfunction, or obstruction at any part of the lacrimal drainage apparatus. Appositional eyelids abnormalities and poor pump function are usually bilaterally present in elderly patients and are conveniently recognized. Unilateral epiphora is strongly associated with lacrimal drainage obstruction and must be identified as an important indication for timely la crimal drainage workup^(1)^.

In this report, the authors describe the case of a patient with chronic unilateral epiphora that had been neglected for several years; the patient as prescribed lacrimal system workup because of acute dacryocystitis. Following a diagnosis of nasolacrimal duct obstruction and with an indication for surgery, intraoperative abnormal lacrimal sac mucosa was subjected to biopsy, and a diagnosis of squamous cell carcinoma was established.

## CASE REPORT

A 63-year-old man with an 8-year history of unilateral epiphora in the right eye following acute ipsilateral inflammation of the medial canthal region was treated with short-term systemic antibiotic therapy and referred for specialized evaluation. His medical history was unremarkable.

Visual acuity was 20/20 in each eye, and the results of the slit-lamp examination were unremarkable. In the right eye, a palpable firm mass below the medial canthal tendon showed no expression of fluid from the puncta. The overlying skin was erythematous, and the patient reported moderate pain. “Hard stop” was appreciated during probing of the canaliculi, and saline irrigation did not reach the oral cavity with reflux through the opposite canaliculus. A hypothesis of lower lacrimal drainage system was established, and dacryocystorhinostomy (DCR) indicated.

**Figure 1:**
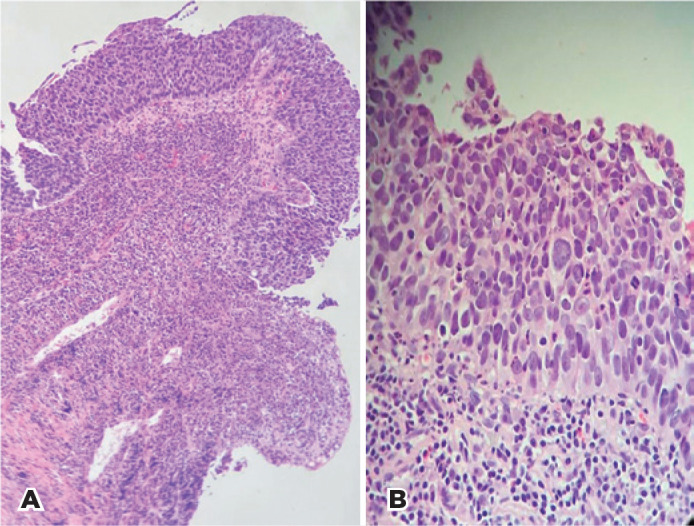
A) Squamous carcinoma, non-invasive type cancer with full thickness atypia. Hematoxylin and Eosin (HE) 100x. B) Dysplastic keratinocytes with marked atypia, abnormal cells, and mitosis. No evidence of invasion. HE 400x.

Intraoperatively, abnormal lacrimal sac mucosa was noted and brownish dacryoliths were found inside the sac. Owing to a suspicion of malignancy, a biopsy of the mucosa was performed. Histopathological examination revealed a disarranged architecture with atypical epithelial cells, including nuclear hyperchromasia and numerous atypical mitotic figures (Figure 1A and 1B). Immunohistochemistry shoed that the cells were positive for p63 protein. Based on these findings, we established a diagnosis of squamous cell carcinoma (SCC) *in situ*.

**Figure 2:**
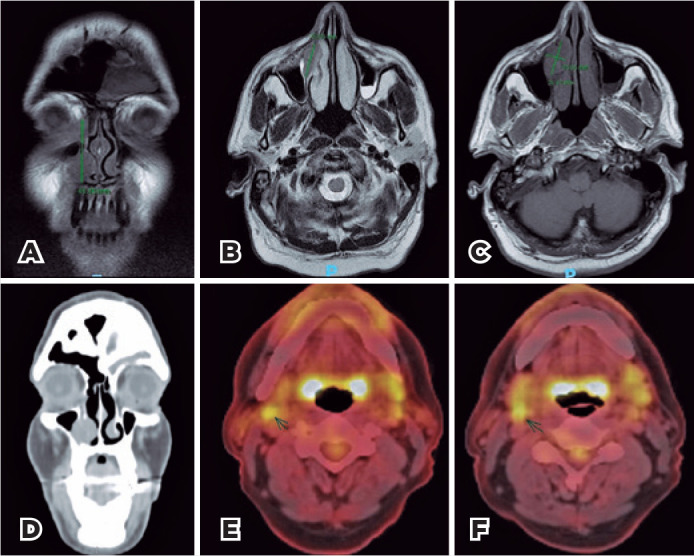
A) Coronal T1-weighted magnetic resonance imaging (MRI) demonstrates infiltrative lesion with isosignal. B) Axial T2-weighted MRI demonstrates infiltrative lesion with slight hypersignal. C) Axial T1-weighted fat saturated post gadolinium MRI demonstrates infiltrative lesions with intense contrast enhancement. D) Coronal image, computed tomography (CT), soft tissue window demonstrates infiltrative lesion with soft tissue density. E, F) Axial Positron emission tomography with CT scan (PET-CT) images shows increased FDG uptake of two lymph nodes in the upper right jugular chain (E- slightly higher and F slightly lower).

Further, magnetic resonance imaging (MRI) revealed a 4.2 x 2.6 x 1.5-cm ill-defined mass occupying the lacrimal sac fossa and the nasolacrimal duct, located between the medial wall of the maxillary sinus and the inferior nasal concha (Figure 2A-2C); same findings were observed in the tomographic images obtained using positron emission tomography with computed tomography (CT) scan positron emission tomography (PET-CT) (Figure 2D). The lesion showed isosignal on T1-weighted images, slight hypersignal on T2-weighted images, and intense contrast uptake. PET-CT demonstrated involvement of two lymph nodes in the upper right jugular chain (Figures 2E-2F).

**Figure 3:**
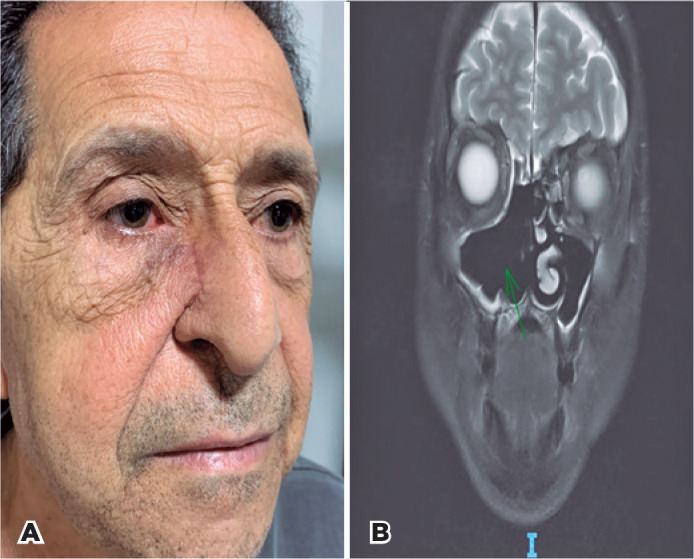
(A) Aspect of the patient after tumor resection surgery and adjuvant radiotherapy. (B) Coronal T2-weighted magnetic resonance imaging (MRI) demonstrated resection of the medial wall of the maxillary sinus and right nasal turbinates.

Extended tumor excision with partial right maxillectomy and sinusotomy through lateral rhinotomy was performed. Additionally, 30 sessions of 60 Gy radiotherapy were performed locally and at the cervical area. At the time of writing this report, the patient has remained recurrence-free for 2 y after the procedure (Figure 3A and B).

## DISCUSSION

Most patients with lacrimal drainage obstruction present with unilateral or bilateral, constant, or intermittent tearing. The main form of dacryostenosis is the idiopathic primary acquired nasolacrimal duct obstruction that is most commonly observed in middle-aged or elderly female patients. Secondary causes include inflammatory, infectious, traumatic, mechanical, or neoplastic causes^(2)^. Neoplasms that result in chronic nasolacrimal duct obstruction are uncommon but not rare. Of these, >50% are malignant, with a distinction between epithelial and non-epithelial tumors^(3)^.

Epithelial tumors represent more than two-thirds of all lacrimal sac malignant tumors and include SCC, transitional cell carcinoma, adenocarcinoma, mucoepidermoid tumors, and adenoid cystic carcinoma, where SCC is the most common type^(4)^. Lacrimal sac malignances tend to be locally invasive, metastasize, and have a high recurrence rate^(3)^. Kuo et al. performed a review of 10 patients with primary sac malignancies and reported a metastasis rate of 50% and a mortality rate of 50%^(5)^. Song et al. reported a 5-year overall survival of 88%^(4)^.

The management of lacrimal sac tumors is primarily based on retrospective case series, and the most appro priate treatment strategy remains controversial. Conventional treatment methods include wide surgical resection followed by radiation therapy and/or chemotherapy^(4-6)^. Tumors with extension beyond the sac may require more aggressive surgeries, including exenteration, sinus resection, and cervical lymph node removal^(4)^. In order to shrink and concrete the tumor preoperatively, the administration of radiotherapy, and chemotherapy before the surgery has recently been recommended^(6)^. In our case, we performed extended *en bloc* tumor resection associated with postoperative adjuvant radiotherapy to prevent locoregional recurrence. We achieved a suitable functional and aesthetic result, with the preservation of the eye and vision with no recurrence till 2 y postoperatively.

The main symptoms of lacrimal sac tumors at diagnosis are unilateral tearing and the presence of a palpable mass, especially if extending above the medial canthal tendon^(3-6)^. Less commonly, recurrent dacriocystitis^(3,4)^, bloody tears^(3)^ and pain^(6)^ may occur. Owing to its rarity, nonspecific symptoms, and low index of suspicion, there is an average 3-year delay in diagnosing lacrimal drainage system malignancies^(3)^. In our report, the patient had symptoms of tearing for 8 y before the diagnosis, even after several ophthalmologist visits. It is important to emphasize that the epiphora is found in about 53%59% of patients at the time of diagnosis^(3,4)^; however, its absence does not exclude the diagnostic possibility because early tumors, both malignant and benign, may present clinically with the patent lacrimal pathway.

Some authors recommend that preoperative lacrimal imaging be reserved for major craniofacial anomalies, occasional failed DCR, or signs of tumor^(7)^. In our case, the sign of a palpable medial canthus mass without regurgitation of mucus should have indicated preoperative imaging, thus abbreviating the tumor diagnosis.

Routine surgical biopsy of the lacrimal sac in DCR is not a consensus among surgeons. Certain authors argue that some neglected pathological processes could be detected^(3,8)^, while others suggest biopsy only for suspicious cases^(9,10)^. In our practice, lacrimal sac biopsy specimens are not routinely submitted for pathologic examination during DCR. In this case, an abnormal-appearing lacrimal sac was noted during the surgery and malignancy was conveniently diagnosed after the biopsy.

This report demonstrates the importance of a thorough evaluation for identifying the underlying cause of tearing, especially in patients with chronic unilateral presentation. Although neoplasia is an uncommon cause of nasolacrimal obstruction, ophthalmologists should be aware of the signs of this disorder because delayed diagnosis may worsen the prognosis.
